# Influences of Ar Flow-Rate and Sublimation Temperature on the Sublimation Product of Analytical Reagent MoO_3_

**DOI:** 10.3390/molecules30132751

**Published:** 2025-06-26

**Authors:** Feng-Jiao Du, Jian-Jun Yu, Jian-Gang Liu, Lu Wang

**Affiliations:** 1 College of Mechanical and Electrical Engineering, Wuyi University, Wuyishan 354300, China; 2 Key Laboratory for Ferrous Metallurgy and Resources Utilization of Ministry of Education, Wuhan University of Science and Technology, Wuhan 430081, China; 3 Hubei Provincial Key Laboratory for New Processes of Ironmaking and Steelmaking, Wuhan University of Science and Technology, Wuhan 430081, China

**Keywords:** β-MoO_3_, α-MoO_3_, Ar, Sublimation temperature, formation mechanism

## Abstract

In this work, the influences of the Ar flow-rate and sublimation temperature on the phase composition and morphological structure of the sublimation products of analytical reagent MoO_3_ are investigated. The results show that the sublimation products are always composed of thermodynamically stable orthorhombic molybdenum trioxide (α-MoO_3_) and metastable monoclinic molybdenum trioxide (β-MoO_3_) under different reaction conditions, among which the proportion of β-MoO_3_ gradually increases with the increase in Ar flow-rate and the decrease in sublimation temperature. The formation temperature of α-MoO_3_ is mainly between 780 K and 847 K, with the particles exhibiting an obvious sheet-like morphology. This work also finds that β-MoO_3_ is mainly generated below 500 K; however, due to the co-actions of the deposition of gaseous MoO_3_ molecules, the adsorption of Ar molecules, and the collision effect between the different particles, the newly formed β-MoO_3_ is more inclined to take a spherical-shaped morphology in order to maintain its lowest energy state.

## 1. Introduction

Molybdenum trioxide (MoO_3_) possesses multiple excellent physicochemical properties; hence, it is widely used in numerous contexts, such as batteries [1,2], catalysts [3,4], sensors [5,6,7], and supercapacitors [8,9]. Additionally, as an important intermediate material, MoO_3_ is usually used to prepare ultrafine Mo powder [10,11], molybdenum carbide [12], and molybdenum disulfide [13,14,15]; thus, its study has attracted significant attention in recent years [16,17]. In general, MoO_3_ has four kinds of crystal types, and the different types have different crystal structures and physicochemical properties. It has been reported [18] that the metastable monoclinic molybdenum trioxide (β-MoO_3_) usually has a better catalytic efficiency and electrochemical performance than that of the thermodynamically stable orthorhombic molybdenum trioxide (α-MoO_3_). Therefore, the large-scale preparation of β-MoO_3_ has received increasing attention in recent decades [19,20,21].

β-MoO_3_ was first prepared by McCarron via a solution method [22]; since then, numerous preparation methods have been proposed [23,24]; however, the issues of high cost and route complexity still exist. The physical vapor deposition (PVD) method has the advantages of low cost and process simplicity and has thus gradually become a common method of preparing micro- and nano-sized powder materials. In view of this, the PVD method has also been used in the preparation of micro-/nano-sized β-MoO_3_ in previous studies [25,26,27], in which water vapor, oxygen, and air atmospheres were adopted, respectively.

As a common gas, Ar is widely used in various industrial fields; however, there have been few studies concerning the preparation of β-MoO_3_. The present study is intended to fill this gap; in this paper, the influences of the Ar flow-rate and sublimation temperature on the phase composition and morphological structure of the sublimation products are illustrated.

The chemical vapor deposition (CVD) reaction between gaseous MoO_3_ and H_2_ for the purpose of preparing ultrafine Mo powder had been reported in our previous work [28]. The results indicated that pure Mo powder can be obtained when the carrier gas (Ar) and reducing gas (H_2_) are 300 and 500 mL/min, respectively; however, under other conditions, ultrafine MoO_2_ powder can also be obtained. Herein, the formation of MoO_2_ was understood to result from the chemical vapor reaction. In our preliminary experiment, carried out before this work, the sublimation–condensation process of MoO_3_ under 1273 K and Ar atmosphere conditions was conducted, and the results indicated that MoO_2_ can also be generated under an extremely high Ar flow-rate. Therefore, in order to further clarify the formation mechanism of different ultrafine powders during the CVD process, the authors carried out the following study.

## 2. Results

### 2.1. XRD for Phase Analysis

**Figure 1:**
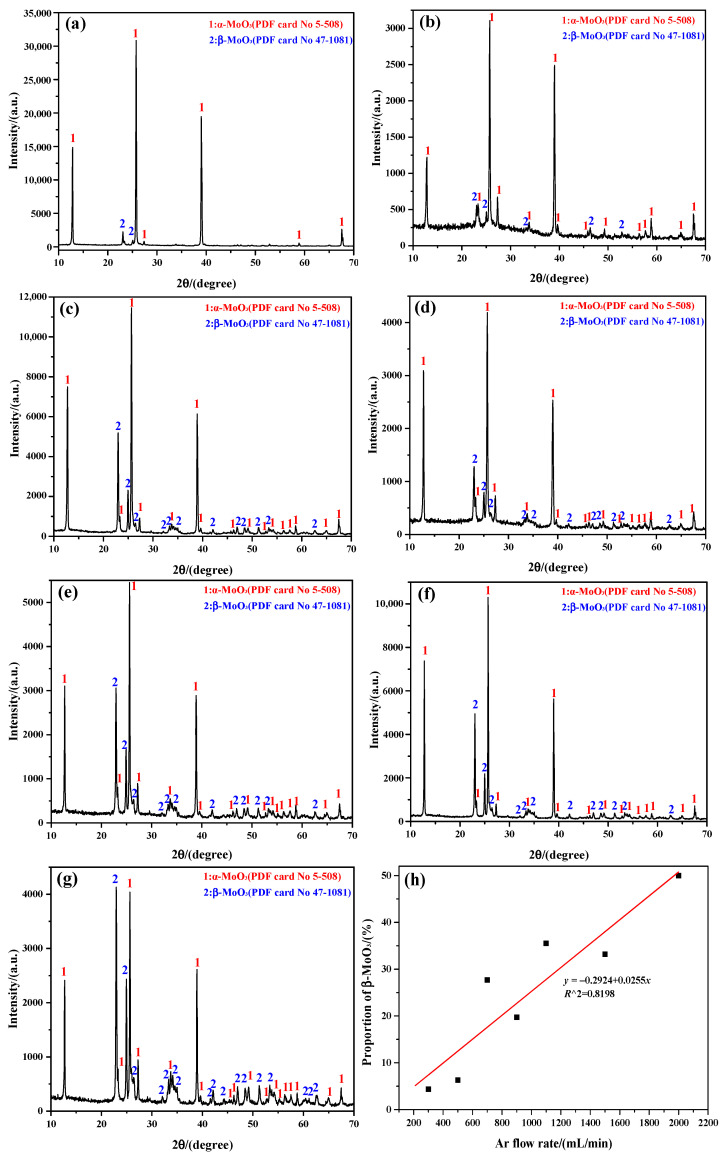
Phase composition of the sublimation products obtained at 1273 K under different Ar flow-rates: (**a**) 300 mL/min; (**b**) 500 mL/min; (**c**) 700 mL/min; (**d**) 900 mL/min; (**e**) 1100 mL/min; (**f**) 1500 mL/min; (**g**) 2000 mL/min. (**h**) The proportion of β-MoO_3_ in the mixed MoO_3_ crystals.

The phase identification of the sublimation products obtained at 1273 K under different Ar flow-rates are mainly analyzed by “Search-Match 2.1.1.0” software, with the corresponding results shown in Figure 1. The results show that the sublimation products always consist of α-MoO_3_ (PDF card No. 5-508; space group: *Pbnm*; lattice parameter: *a* = 3.962 Å, *b* = 13.858 Å, and *c* = 3.697 Å) and β-MoO_3_ (PDF card No. 47-1081; space group: *P21/c*; lattice parameter: *a* = 7.118 Å, *b* = 5.366 Å, and *c* = 5.568 Å), with a sharp and strong diffraction peak, indicating that both have an excellent crystallinity. It can be observed that the peak intensity of β-MoO_3_ increases with the increasing Ar flow-rate, which suggests that the proportion of β-MoO_3_ in the mixed MoO_3_ crystals gradually increases. Herein, the proportion of β-MoO_3_ is obtained by calculating the ratio of the peak area of β-MoO_3_ to that of both α-MoO_3_ and β-MoO_3_ according to the XRD pattern, using the common “Origin” software for the calculation. To be more specific, when the Ar flow-rates are 300, 500, 700, 900, 1100, 1500, and 2000 mL/min, the proportion of β-MoO_3_ in the mixed MoO_3_ crystals is 4.35, 6.29, 27.69, 19.73, 35.53, 33.18, and 49.96%, respectively; that is, the proportion of β-MoO_3_ gradually increases with the increase in the Ar flow-rate, even if some deviations exist, as illustrated in Figure 1h. Because a partial overlap exists between the diffraction peaks of the two MoO_3_ crystals, the calculation results of their respective peak areas may not be very accurate; therefore, the relationship between the proportion of β-MoO_3_ and the Ar flow-rate does not seem to indicate a strong correlation. Even so, the calculated deviation parameter *R*^2^ is still up to 0.8198, and the fitting equation can be written as *y* = −0.2924 + 0.0255*x*, where *y* denotes the proportion of β-MoO_3_ in the mixed MoO_3_ crystals(%), and *x* presents the Ar flow-rate (mL/min).

**Figure 2:**
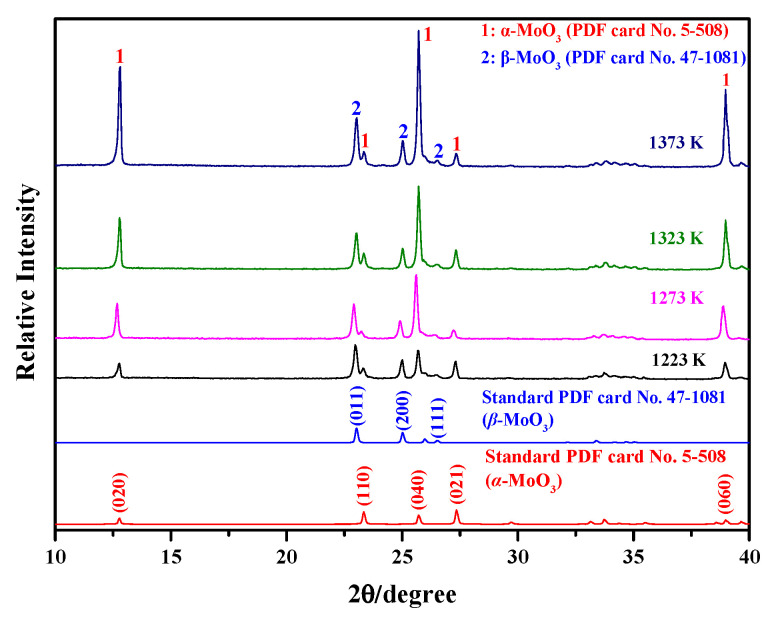
Phase composition of the sublimation products obtained under different sublimation temperatures (Ar flow-rate: 1100 mL/min).

When the Ar flow-rate is 1100 mL/min, the influence of the sublimation temperature on the phase composition of the sublimation products is also analyzed. Herein, in order to allow for the diffraction peaks to be seen more clearly, only scanning angles of 10–40° are selected. Meanwhile, the standard PDF card patterns for α-MoO_3_ (No. 5-508) and β-MoO_3_ (No. 47-1081) are also provided. The corresponding results are shown in Figure 2. The figure shows that the sublimation products contain both α-MoO_3_ and β-MoO_3_, similar to the cases observed under different Ar flow-rates. These results suggest that the change in sublimation temperature has no obvious influence on the phase composition. From Figure 2, it can also be deduced that the crystallinities of the two MoO_3_ species both gradually increase with the increase in sublimation temperature due to the increasing intensity of the strongest diffraction peak. However, the proportion of β-MoO_3_ within the sublimation product gradually decreases. In other words, the proportion of α-MoO_3_ gradually increases with the increase in the sublimation temperature; moreover, due to the relatively strong peaks of the crystal indices of (020), (040), and (060), the newly formed α-MoO_3_, with a certain anisotropy and preferred growth characteristics, can also be deduced.

### 2.2. FESEM for Morphology Observation

**Figure 3:**
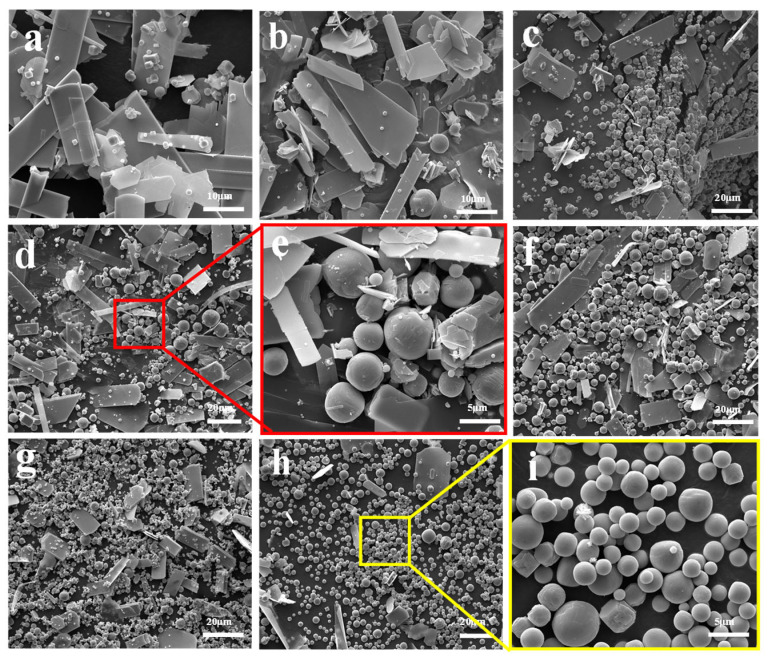
Morphological structure of the sublimation products obtained at 1273 K under different Ar flow-rates: (**a**) 300 mL/min; (**b**) 500 mL/min; (**c**) 700 mL/min; (**d**) 900 mL/min; (**e**) The enlarge image showed in (**d**) marked with red area; (**f**) 1100 mL/min; (**g**) 1500 mL/min; (**h**) 2000 mL/min; (**i**) The enlarge image showed in (**h**) marked with yellow area.

Figure 3 illustrates the morphological structure of the sublimation products obtained at 1273 K under different Ar flow-rates. It shows that the sublimation products mainly include two different morphologies—one is spherical, and the other is platelet-shaped. The results also show that the amount of the spherical particles gradually increases, while that of the platelet-shaped particles gradually decreases with the increase in Ar flow-rate. Combining the results shown in Figure 1 and those in the literature [27], it can be inferred that the spherical particle is β-MoO_3_ and the platelet-shaped particle is α-MoO_3_. The diameter of the spherical β-MoO_3_ particle is also measured, and the results indicate that it is in the range of 1–5 μm, regardless of the Ar flow-rate. In our previous work [27], the diameter of the spherical β-MoO_3_ was found to gradually decrease with the increase in the pumping speed of the vacuum pump, with the average diameter being about 0.25 μm. The deviation may result from the difference in the flow-rate of the gaseous molecules. In our previous work, the flow-rate of the gaseous molecules was as high as 40 L/min; while in the current work, the Ar flow-rate is only in the range of 300–2000 mL/min. Due to the small and narrow range of the Ar flow-rate, the particle diameter of the spherical β-MoO_3_ prepared in this work seems to be unchanged. Combining the previous and current results, this work suggests that the reasonable regulation of the Ar flow-rate during the sublimation process is important for controlling the particle dimension of the spherical β-MoO_3_ powder.

**Figure 4:**
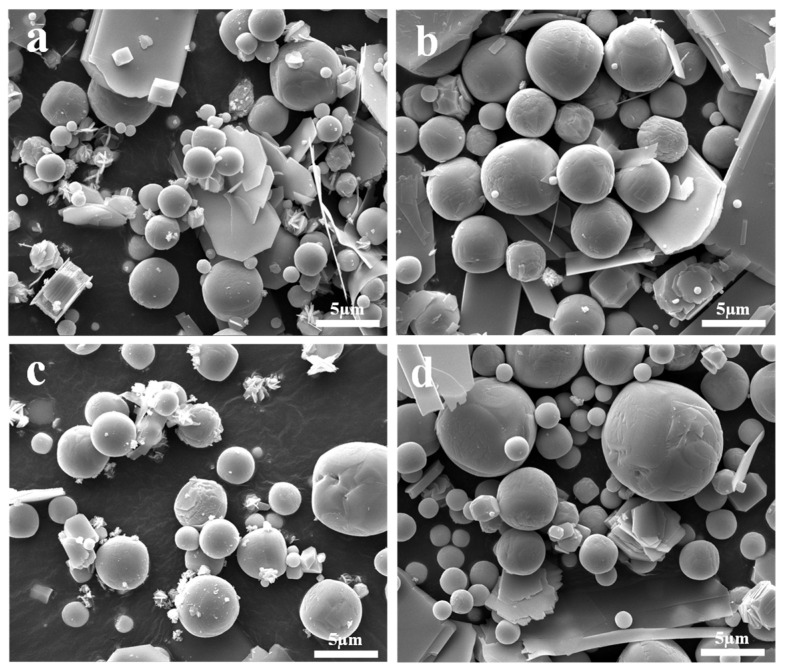
Morphological structure of the sublimation products obtained under different sublimation temperatures: (**a**) 1223 K; (**b**) 1273 K; (**c**) 1323 K; (**d**) 1373 K. (Ar flow-rate: 1100 mL/min).

Figure 4 presents the morphological structure of the sublimation products obtained under different sublimation temperatures. It shows that the sublimation products also exhibit two distinct morphologies, namely, platelet-shaped α-MoO_3_ and spherical β-MoO_3_. Similar to the results observed under different Ar flow-rates, the diameter of the spherical particles is also in the range of 1–5 μm; however, due to the fast growth rate of particles at a higher temperature, the number of spherical particles with large dimensions gradually increases with the increase in the sublimation temperature.

## 3. Discussion

**Figure 5:**
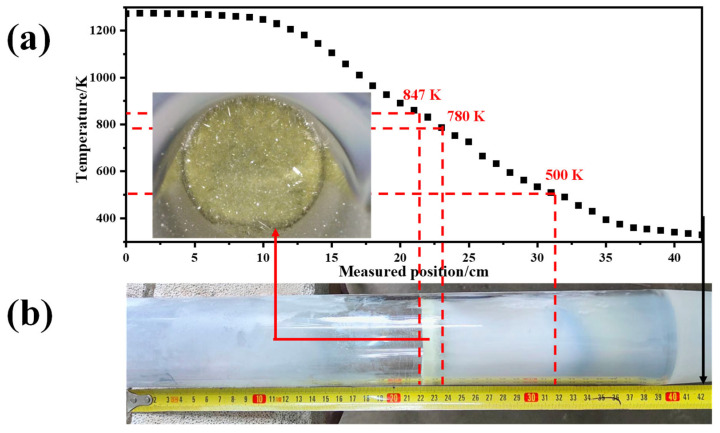
Macroscopic results of the current experiment: (**a**) Temperature distribution in the quartz tube at the temperature of 1273 K; (**b**) Image of the used quartz tube after the experiment.

MoO_3_ has a strong sublimation property, and its sublimation rate can be very large, even at 973 K [29,30,31]. When solid MoO_3_ raw material is in the high-temperature-zone of the Si-C electronic furnace, it will sublimate into gaseous forms, in which the gaseous (MoO_3_)*_n_* (*n* = 2, 3, 4, and 5) species dominate [32,33]. Due to the continuous introduction of carrier gas (Ar), the newly formed gaseous MoO_3_ species will be rapidly carried through the quartz tube toward the collection bottle. During this process, the temperature of the gaseous MoO_3_ gradually decreases, which can be seen in the temperature distribution diagram in the quartz tube, as shown in Figure 5a. It has been reported [22,34] that when the temperature is below 723 K, gaseous MoO_3_ is preferentially condensed into β-MoO_3_, while when the temperature exceeds that value, α-MoO_3_ is preferentially formed. Because the temperature of the collection bottle is lower than 373 K, β-MoO_3_ will form in the collection bottle. The larger the Ar flow-rate, the more gaseous MoO_3_ there will be, and the higher the relative content of deposited β-MoO_3_ in the collection bottle will be. Meanwhile, a certain amount of gaseous MoO_3_ will condense on the wall surface of the quartz tube, which is beneficial for the formation of α-MoO_3_ due to its relatively high temperature. On the one hand, α-MoO_3_ has an orthorhombic crystal structure with a double-layered network of [MoO_6_] octahedral. These layers are stacked along the *b*-axis (010) through van der Waals forces. Moreover, the structure is beneficial for the formation of sheet-like images [35]. On the other hand, the newly formed α-MoO_3_ in the quartz tube wall is still easily sublimated, even if its sublimation extent is weakened; in this case, the newly formed gaseous MoO_3_ will transform to the relatively low-temperature zone and condensate on the edge of the original α-MoO_3_ crystals; that is, parts of the volatilization–condensation process are still undertaken in this zone. As a result, α-MoO_3_ is more inclined to form a sheet-like structure at a high temperature. One thing that must be noted is that when the α-MoO_3_ forms in the high-temperature zone, the Ar airflow will carry parts of the α-MoO_3_ into the collection bottle. Therefore, the sublimation products in the collection bottle always contain both α-MoO_3_ and β-MoO_3_. Even though an increment in the Ar flow-rate would enhance its carrying capacity, this capacity is not sufficient to take away all the sheet-like α-MoO_3_; that is, most of the α-MoO_3_ are still adhered to the wall of the quartz tube, as seen in the inset of Figure 5a. For this reason, even if all the analytical reagent MoO_3_ is completely sublimated, and no residue exists in the alumina crucible, the mass of the sublimation product in the collection bottle will still only be in the range of 1–2 g; that is, the yield is about 20–40%. Herein, the temperature of the α-MoO_3_ deposited on the quartz tube is determined to be in the range of 780 K to 847 K, and that of the β-MoO_3_ is lower than 500 K; in other words, the above temperature ranges denote the stable existence intervals of α-MoO_3_ and β-MoO_3_, respectively. Due to the use of an Ar atmosphere and the lack of O_2_ in the reaction system, as well as the unstable characteristics of β-MoO_3_, a certain number of oxygen vacancies will form in the β-MoO_3_ crystal during the condensation process [36], and this is the reason why the colors of the β-MoO_3_ deposited on different sites differ, as shown in Figure 5b.

**Figure 6:**
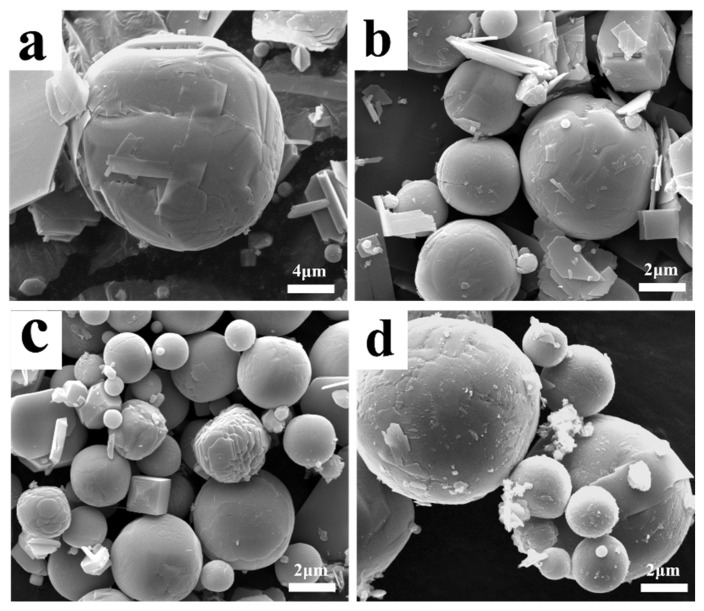
Some special morphologies of the sublimation product obtained under different conditions: (**a**) 1273 K, 500 mL/min Ar; (**b**) 1273 K, 900 mL/min Ar; (**c**) 1223 K, 1100 mL/min Ar; (**d**) 1323 K, 1100 mL/min Ar.

Figure 6 depicts some special morphologies of the sublimation products obtained under different conditions. Because spherical particles have the lowest surface free energy, and because the rapid reduction in the temperature of gaseous MoO_3_ would increase its saturated vapor pressure, in order to maintain the lowest energy state, numerous spherical particles will form during the condensation process. However, as shown in Figure 6, some newly formed particles are not perfectly spherical-shaped, and several transition states, such as cubic or elliptic particles, with many high-energy sites on the surface, also exist. In this case, the subsequent gaseous MoO_3_ molecules can be deposited on these sites to fill the voids and to make the particle spherical-shaped. In addition to gaseous MoO_3_ molecules, the small volume ratio of Ar molecules may also play important roles. When Ar molecules are adsorbed on the surface of MoO_3_, they can average the surface energy anisotropy, which may cause any oriented plane to hold the same surface energy and, finally, produce a spherical crystal. This is to say, that the gas molecules’ adsorption is an easy way to manipulate the crystal surface energy [37], which defines the notable Wulff shape of a single crystal. From this figure, it can also be observed that some small or platelet-shaped particles are adsorbed on the surface of the larger ones; in these cases, as time goes on, these small or platelet-shaped particles may gradually disappear and become a part of the larger one, as supported by the clear crystal boundary on the crystal surface, and this process is consistent with the classical Ostwald ripening mechanism [38]. Therefore, due to the co-action of the continuous deposition of gaseous MoO_3_ molecules, the adsorption of Ar molecules, and the particle collision effect, large spherical particles will form.

**Figure 7:**
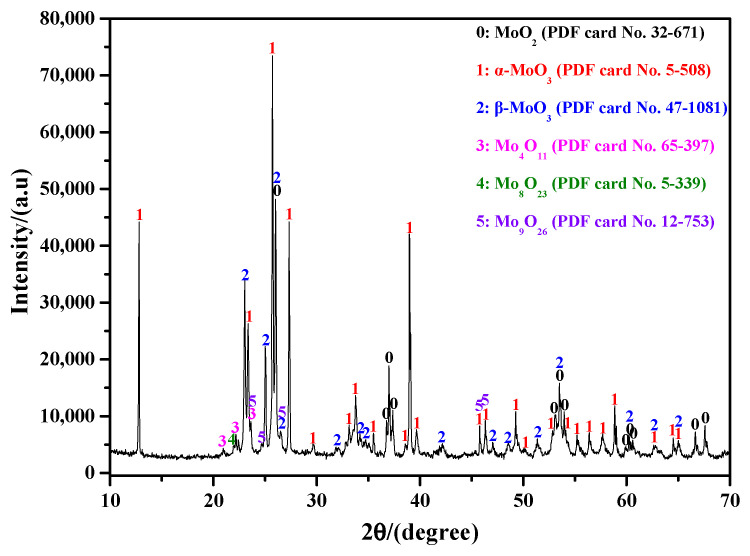
XRD result of the sublimation product of analytical reagent MoO_3_ under the Ar atmosphere with an extremely high flow-rate (the condition was realized via the vacuum pump).

Table 1 lists some preparation methods for β-MoO_3_ powder. It shows that many parameters, such as Mo source, sublimation temperature, and reaction atmosphere, may have close relationships with the microstructure of β-MoO_3_. However, it is also the case that β-MoO_3_ is entirely formed at a relatively low temperature [25,26,27]. Since the Ar gas is used in this work, many oxygen vacancies form inside the MoO_3_ species. When the Ar flow-rate is not very high, metastable β-MoO_3_ can be obtained, while when the Ar flow-rate is sufficiently high, the oxygen content in the reaction system will be extremely low; meanwhile, the condensation rate of the gaseous MoO_3_ will rapidly increase, which will lead to the formation of more oxygen vacancies. In this situation, the newly formed metastable β-MoO_3_ will be decomposed into numerous sub-oxides, such as MoO_2_, Mo_4_O_11_, and other oxides; this is the reason why many other sub-oxides appeared in our preliminary finding, as shown in Figure 7. Therefore, based on the current results, and those reported in our previous study, we propose that increasing the sublimation temperature appropriately may be a good option for improving the preparation efficiency of ultrafine β-MoO_3_ powder; moreover, the reasonable control of the reaction atmosphere and its gas flow-rate are also very important.

## 4. Materials and Experimental Procedures

### 4.1. Materials

**Figure 8:**
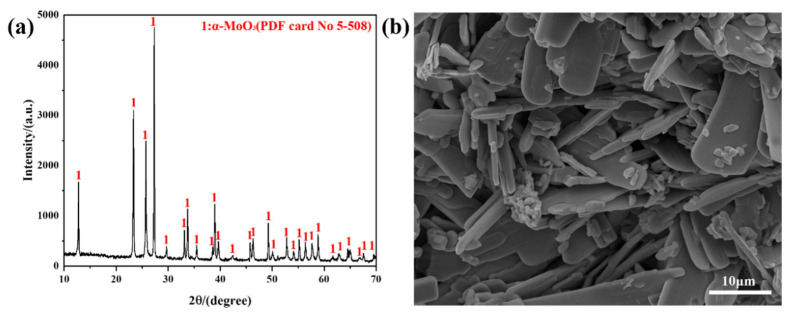
Raw material used in the work: (**a**) XRD pattern; (**b**) FESEM micrograph.

Analytical reagent MoO_3_ (AR, 99.5%), purchased from Shanghai Aladdin Biochemical Technology Co., Ltd., is used as a raw material. The XRD result shows that the raw material belongs to α-MoO_3_, and FESEM imaging shows that the raw material exhibits a sheet-like morphology, as observed in Figure 8. The carrier gas used in the work is high-purity Ar (the amount of impurities < 5 ppm).

### 4.2. Experimental Procedures

**Figure 9:**
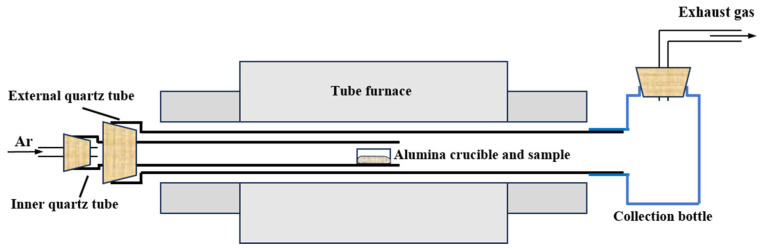
Schematic diagram of the experimental device.

Figure 9 shows the schematic diagram of the experimental device. In each of the experiments, 5 g of MoO_3_ raw material was weighed and placed into an alumina crucible (50 mm × 20 mm × 20 mm). Then, the MoO_3_-containing crucible was placed on the right end of the inner quartz tube (inner diameter: 30 mm; outer diameter: 38 mm). After the Si-C electronic furnace reaching the specified temperature, the inner quart tube was inserted into the external quartz tube (inner diameter: 50 mm; outer diameter: 60 mm), with the MoO_3_ raw material placed into the constant-temperature zone of the furnace. Meanwhile, Ar gas with a certain flow-rate was introduced from the left end of the inner quartz tube to carry the gaseous MoO_3_ towards the collection bottle and accelerate its condensation rate. After 1 h of reaction time, we turned off the Ar gas and then collected the sublimation product in the collection bottle for further testing. In order to ensure parity with our previous work [28] and make a further contribution under the same study conditions, Ar flow-rates of 300, 500, 700, 900, 1100, 1500, and 2000 mL/min and sublimation temperatures of 1223, 1273, 1323, and 1373 K were selected for the current experiments.

### 4.3. Product Characterization

The phase composition of the sublimation product was analyzed by an X-ray diffraction analyzer (XRD; D8 Advance, AXS Corporation, Bruker, Germany; operation voltage: 30 kV; operation current: 30 mA; scanning speed: 10°/min; scanning angle: 7–90°; Cu-Kα1 radiation with the wavelength of 1.54056 Å), and its morphological structure was observed via field emission scanning electron microscopy (FESEM; Nova 400 Nano SEM, FEI Corporation, Hillsboro, OR, USA; Operation voltage: 15 kV).

## 5. Conclusions

In this work, the phase composition and morphological structure of the sublimation products of analytical reagent MoO_3_ are investigated. The following conclusions are drawn.

(1) The sublimation products obtained under different Ar flow-rates and sublimation temperatures are always composed of both α-MoO_3_ and β-MoO_3_, among which the proportion of β-MoO_3_ gradually increases with the increase in Ar flow-rate and the decrease in sublimation temperature.
(2) Platelet-shaped α-MoO_3_ is usually generated in the range of 780 K to 847 K, while spherical-shaped β-MoO_3_ forms below 500 K. The diameter of β-MoO_3_ is determined to be in the range of 1–5 μm and has no obvious relationship with the Ar flow-rate due to its low value and narrow range.
(3) Due to the co-actions of the deposition of gaseous MoO_3_ molecules, the adsorption of Ar molecules, and the particle collision between different particles, the newly formed β-MoO_3_ particles usually exhibit a spherical-shaped morphology in order to decrease their surface free energy.

## Figures and Tables

**Table 1:** Different preparation methods for ultrafine β-MoO_3_ powder.

References	Preparation Processes	Images	Microstructures
Sun et al. [25]	$\begin{array}{l} \mathrm{High}-\mathrm{grade}\;\mathrm{molybdenite}\frac{873\;K}{2h}{\mathrm{MoO}}_{3}\frac{\mathrm{water}\;\mathrm{vapor}\;\mathrm{atmosphere},\;300\;L/h}{1173-1373\;K}\\ {\alpha -\mathrm{MoO}}_{3}{(973\;K)+\beta -\mathrm{MoO}}_{3}\left(473\;K\right) \end{array}$	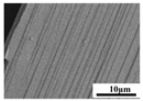	The sample deposited at 973 K, presenting as thin strips, was pure α-MoO_3_, and that deposited at 473 K took the form of spherical β-MoO_3_ and thin-strips α-MoO_3_
		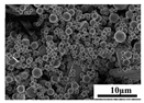	
Ngo et al. [26]	${\alpha -\mathrm{MoO}}_{3}\;\mathrm{powder}\;\left(99.9\%\right)\frac{8{\;L/\min\;O}_{2}}{1023\;K,30\;\min}{\alpha -\mathrm{MoO}}_{3}{\;+\;\beta -\mathrm{MoO}}_{3}$	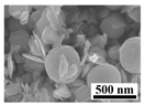	Composed of large spherical particles with sizes of about 200 nm; some needle-shaped particles also exist
Wang et al. [27]	${\mathrm{Industrial}\;\mathrm{MoO}}_{3}\frac{\mathrm{air},\;1373\;K,\;\mathrm{water}\;\mathrm{cooling}}{\mathrm{pumping}\;\mathrm{speed}:\;40\;\mathrm{cm}/s}{\alpha -\mathrm{MoO}}_{3}{\;+\;\beta -\mathrm{MoO}}_{3}$	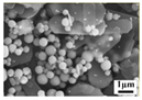	The obtained product is composed of spherical β-MoO_3_ and platelet-like α-MoO_3_ particles
Mariotti et al. [39]	$\;\mathrm{Mo}\;\mathrm{wire}\frac{\mathrm{patch}\;\mathrm{antenna}-\mathrm{based}\;\mathrm{atmospheric}\;\mathrm{microplasma}\;\mathrm{processing}}{\mathrm{Si}(100)\;\mathrm{substrate}\;\mathrm{with}\;a\;\mathrm{conducting}\;\mathrm{Pt}\;\mathrm{layer}\;(\mathrm{Si}-\mathrm{Pt}\;\mathrm{substrate})}{\beta -\mathrm{MoO}}_{3}$	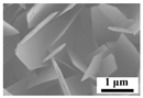	The obtained β-MoO_3_ is a nanosheet with a thickness of 50–100 nm
This work	${\mathrm{MoO}}_{3}\left(\mathrm{AR},99.5\%,\;\mathrm{Aladdin}\right)\frac{1100\;\mathrm{mL}/\min}{1273\;K}{\alpha -\mathrm{MoO}}_{3}{\;+\;\beta -\mathrm{MoO}}_{3}$	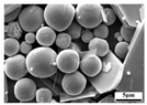	The sublimation products include two different species: spherical β-MoO_3_ and platelet-shaped α-MoO_3_

## Data Availability

The raw data can be provided upon reasonable request.
